# O2 supplementation disambiguation in clinical narratives to support retrospective COVID-19 studies

**DOI:** 10.1186/s12911-024-02425-2

**Published:** 2024-01-31

**Authors:** Akhila Abdulnazar, Amila Kugic, Stefan Schulz, Vanessa Stadlbauer, Markus Kreuzthaler

**Affiliations:** 1 Institute for Medical Informatics, Statistics and Documentation, Medical University of Graz, Graz, Austria; 2 CBmed GmbH - Center for Biomarker Research in Medicine, Graz, Austria; 3 Division of Gastroenterology and Hepatology, Department of Internal Medicine, Medical University of Graz, Graz, Austria

**Keywords:** Natural language processing, Machine learning, Deep learning, Electronic health records, COVID-19

## Abstract

**Background:**

Oxygen saturation, a key indicator of COVID-19 severity, poses challenges, especially in cases of silent hypoxemia. Electronic health records (EHRs) often contain supplemental oxygen information within clinical narratives. Streamlining patient identification based on oxygen levels is crucial for COVID-19 research, underscoring the need for automated classifiers in discharge summaries to ease the manual review burden on physicians.

**Method:**

We analysed text lines extracted from anonymised COVID-19 patient discharge summaries in German to perform a binary classification task, differentiating patients who received oxygen supplementation and those who did not. Various machine learning (ML) algorithms, including classical ML to deep learning (DL) models, were compared. Classifier decisions were explained using Local Interpretable Model-agnostic Explanations (LIME), which visualize the model decisions.

**Result:**

Classical ML to DL models achieved comparable performance in classification, with an F-measure varying between 0.942 and 0.955, whereas the classical ML approaches were faster. Visualisation of embedding representation of input data reveals notable variations in the encoding patterns between classic and DL encoders. Furthermore, LIME explanations provide insights into the most relevant features at token level that contribute to these observed differences.

**Conclusion:**

Despite a general tendency towards deep learning, these use cases show that classical approaches yield comparable results at lower computational cost. Model prediction explanations using LIME in textual and visual layouts provided a qualitative explanation for the model performance.

**Supplementary Information:**

The online version contains supplementary material available at 10.1186/s12911-024-02425-2.

## Background

In January 2020, the World Health Organisation declared a global health emergency based on growing case reports of the novel severe acute respiratory syndrome coronavirus 2 (SARS-CoV-2) [1], leading to the outbreak of Coronavirus disease (COVID-19). The COVID-19 pandemic has created a widespread impact all over the world, with 700 million reported cases and 6 million estimated deaths [2] by late 2023. Up until now, an up-to-date picture of the clinical situation, capable of comparing patient data for a better understanding of all aspects of the disease, has however been impaired by the lack of access to patient data and their lack of standardization, particularly when locked within narrative EHR (electronic health record) content. The ongoing practice of documenting even crucial facts about critically ill patients as free text is a major barrier to the adoption of novel information extraction methods for health care and research. The manual extraction of specific information, such as diagnoses, symptoms, medications, dates, and patient demographics from clinical narratives is a time-consuming and tiresome process. It would have to be done by clinicians familiar with the domain, who would be urgently needed for healthcare delivery in a pandemic context. This motivates the importance of computerised methods to interpret clinical narratives and to extract structured and meaningful information. Text classification with Natural Language Processing (NLP) has reduced the manual time required for analysing clinical text data. However, it is essential to customise the components to fit the specific use case in advance.

### Classical and deep learning approaches

**Text Classification.** A comprehensive analysis of text classification models, spanning classical to DL approaches, highlights the advantages of DL in automatically generating meaningful representations for text mining. However, it also acknowledges limitations, such as neglecting natural sequential and contextual information [3, 4]. Classical ML approaches such as Support Vector Machines (SVMs) have the advantage that their off-the-shelf implementations can not only be trained much faster when compared to deep neural networks [5] but have also an overall better runtime performance. Disambiguation of clinical abbreviations is another essential information extraction task, due to their abundance in clinical narratives, as demonstrated by Jaber and Martínez [6], who used a one-fits-all classifier based on deep learning (DL) models. Many other studies demonstrated the benefit of classical to deep ML algorithms in various healthcare use cases [7–11].

**Machine learning for COVID-19.** COVID-19-related information extraction has covered a broad range of methods, from classical ML to DL models. Daher et al. [12] elaborated on the requirement for supplemental oxygen for admitted patients. Several research works predicted the requirements of oxygen and oxygen therapies in COVID-19 patients using ML approaches [13–16]. Prediction of COVID-19 mortality rates used gradient boosting [17], decision trees [18], artificial neural networks [19] and DL models [20]. Additionally, studies on severity score prediction [21] leveraged explainable artificial intelligence (XAI) approaches [22].

Several factors that contribute to advantages of classical ML approaches are (i) data size and complexity because DL models generally require large amounts of data to learn complex hierarchical representations; (ii) intensive computational resources required for DL models, (iii) the tendency of DL towards overfitting, especially when the dataset is small, and finally (iv) problem-specific considerations, (e.g., scalability, noise and outliers, ethical constraints, user requirements, etc.) can influence the performance of different models.

### O2 saturation in EHRs

A precise understanding of how oxygenation information is recorded in EHRs is essential in retrospective COVID-19 studies. The details of the fraction of inspired oxygen (FiO2), partial pressure of oxygen (PaO2/PO2) and arterial oxygen saturation (SaO2) require attention. FiO2 is 0.21 in room air and increases with supplemental oxygen [23]. PaO2 is sensitive but lacks specificity for gas exchange. The sigmoid oxygen dissociation curve relates PaO2 and SaO2, representing haemoglobin oxygen saturation. Interpreting clinical data, including FiO2, PaO2, and SaO2, is complicated, so accurately extracting information from narratives requires distinguishing and harmonizing related terms and conflicting results [24]. One of the primary challenges lies in the diversity of medical records and the multitude of abbreviations employed, which are often context-dependent and vary across institutions and even between clinicians. The same abbreviation may carry different meanings in distinct contexts and settings. Information extraction systems therefore need to take this ambiguity into account, as well as the existence of synonyms, variants and typos in clinical texts.

**Fig. 1:**
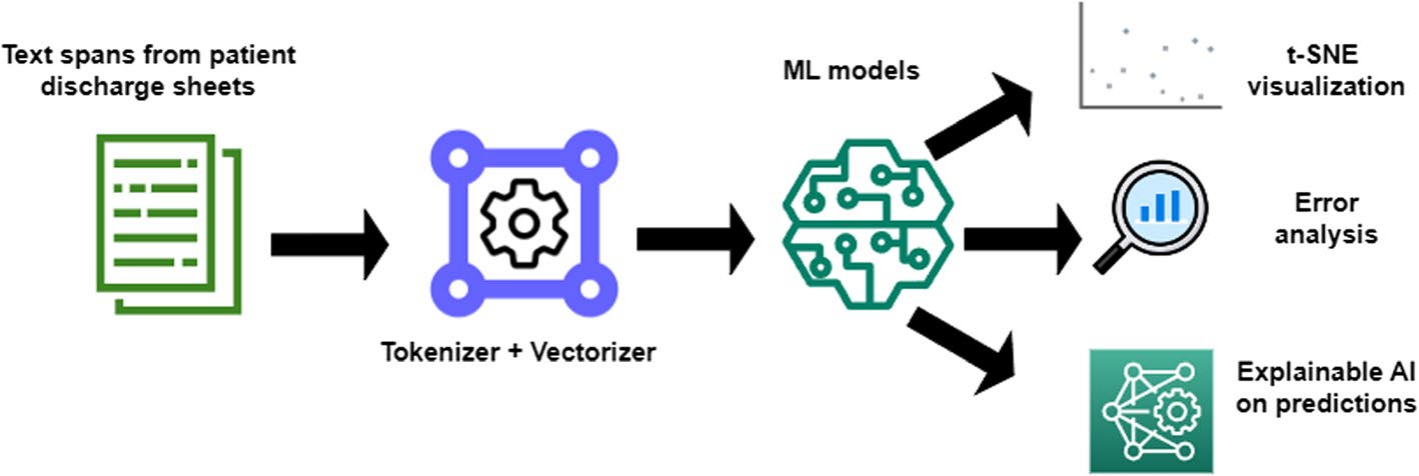
Overview of the proposed methodology encompassing text lines preprocessing, binary text classification, t-SNE visualization, error analysis, and LIME explanation

The focus of our work is on the use of unstructured data on oxygen status and supplementation of COVID-19 patients. The supply of the organism with oxygen is of vital importance, particularly in case of respiratory infection. Current practices involve monitoring of PaO2 and SaO2 using pulse oximetry as a common non-invasive tool [25]. A spontaneous fall in oxygen saturation levels, known as “silent hypoxemia”, is cardinal because low oxygen levels indicate the severity of the disease and predict poor outcomes [26]. Peripheral oxygen saturation (SpO2) determines whether room air oxygen is no longer sufficient and supplementation of oxygen via masks, nasal cannulas or ventilators is required, which often requires intensive care treatment [27]. SpO2/FiO2 ratio is a reliable tool for hypoxemia screening among patients admitted to the emergency departments, particularly during the SARS-CoV-2 outbreak [23]. In addition, this paper addresses the problem of the lack of structured oxygen status data. The problem is complicated by the fact that one and the same concept, *viz.* oxygen, is on the one hand mentioned as a status variable of the patient and a result of measurement, but on the other hand *supplemental* oxygen is referred to as a treatment administered to the patient. Thus, the word “oxygen” may refer to supplementary oxygen treatment as well as to the measurement of SpO2. Differentiation of the oxygen status was based on the measurements of the supplemental oxygen or indicated features for the supplemental oxygen demand and those without any further information regarding the oxygen requirement. In this investigation, the interpretation of whether the reported oxygen status is with or without the supply of oxygen should be done via an adapted model-based approach, as described in this manuscript. This system should support an expert data curator in the identification of relevant document parts to be processed in the next step. Figure 1 represents the flowchart of the proposed method. In addition to the recognition of mentions of oxygen supplementation, this work adds functionality for data visualisation and a methodology for ML model explainability.

The paper is organised as follows: Materials and methods section describes the data and the different types of classifiers used, Results section compares classifier performances and the computational time needed, along with error analysis and model explanation. The Discussion section compares the results with related work and discusses false positives and false negatives.

## Materials and methods

### Dataset

Text lines from discharge summaries of patients affected by COVID-19 were collected from the EHR system of KAGes, an Austrian network of public hospitals. Text lines up to a length of 30 characters, denoting potential oxygen status information, are extracted to build the dataset using a regular expression^1^. This expression was created in several iterations, supported by a data science specialist experienced in clinical queries. The binary classification task is formulated as follows: (i) there is evidence that the patient got oxygen supplementation at some time during the hospital stay, vs. (ii) there is no evidence that the patient received oxygen supplementation.

#### Gold standard creation

The dataset contained 3,844 anonymised text lines. These were annotated by two annotators independently. Both annotators had biomedical backgrounds, were supported by a guideline and passed a series of training sessions. The third annotator with medical expertise validated the annotations so that they could be used as the ground truth. The inter-annotator agreement was high, as evidenced by a Cohen’s Kappa [28] of 0.859, which indicates a 94% accuracy.

The annotation was based on specific text features for example, “*l/min O2 über Nasenbrille*” (litre per minute oxygen via nasal cannula), “*Sauerstoffbedarf*” (oxygen requirement), “*unter CPAP*” (under continuous positive airway pressure), “*mit RL*” (with room air), “*mit NIV*” (with non-invasive ventilation), etc. Some typical text lines are shown along with their class assignments in Table 1.

**Table 1:** Text spans from the dataset along with their classes and relevant tokens translated. Class “1” means the use of supplemental oxygen at some point of time

Text span	Class	Translation of the relevant tokens
“SpO2 99% **mit 10l O2**”	1	“with 10l O2”
“RR 160/80mmHg, HF 76’, Temp. 38,4^∘^C, SpO2 89% **mit RL**”	0	“with room air”
“keine Dyspnoe, kein Fieber, keine Schmerzen, **kein O2 Bedarf**”	0	“no O2 requirement”
“**1L O2/min. über die Nasenbrille** respiratorisch völlig stabil.”	1	“1L O2/min. via nasal cannula”
“O2 **2l/min** bei Bed.”	1	“2l/min”

Of the 3,844 anonymised text lines, 1,435 clearly described the use of supplemental oxygen at some point in time and were thus assigned to class “1”. The remaining 2,409 text lines were assigned to class “0”, of which 45 text lines did not provide any kind of information regarding supplemental oxygen. The dataset was split into training and test data, with a set of constant random state values and a test set size of 20 per cent. The training data consisted of 3,074 spans, with 769 spans in test data.

Finally, a division into training and testing sets was performed by the ‘train_test_split’ function from scikit-learn [29]. To ensure robust evaluation, multiple train-test splits using different random state values^2^ were done. This process helped mitigate the impact of the initial randomization on model performance and assessed its generalisation ability.

### Machine learning approaches

In ML, classification, in general, is a predictive modelling challenge, in which the model has to predict the category of the input data based on fitting of the training dataset. In particular, the classification of text is an elementary NLP task, which is applied wherever input data contain free text. NLP uses different types of ML methods. The following architectures have been applied for the comparative analysis, motivated by a comparison of popular core neural network architectures and their influence on model performance:

#### Model architectures

##### Support Vector Machine (SVM)

SVM is used for both classification and regression tasks. It uses textual data, which is either represented as a vector or a token in a vector space. SVMs attempt to find a hyperplane that best divides the training data into corresponding classes [30]. We used the Support Vector Classifier (SVC) function from scikit-learn [29].

##### Random Forest (RF)

RF is a classification algorithm based on the principles of decision trees. In RF, the set of attributes is randomly split into many subsets, each of which is used to construct decision trees with a few layers. These decision trees collectively form the ‘forest’. The overall performance is then determined based on the outputs of each tree. This randomness in attribute selection and tree construction helps reduce overfitting and enhances the diversity of the trees in the forest. RF is therefore considered a robust and accurate machine learning algorithm [31]. We used the RandomForestClassifier function from scikit-learn.

##### Long Short-Term Memory (LSTM)

LSTM [32] is a type of recurrent neural network. LSTM networks contain feed-forward networks along with corresponding feedback connections. This makes it distinguishable from other neural networks, as it processes sequences of data points. A single LSTM unit is known as a cell, which consists of an input, output and forget gate. These gates control the flow of data in and out of the cell, along with remembering essential information at random time intervals [33]. We used the Keras [34] library for implementing the model layers. Our model architecture consisted of (i) an embedding layer for word representation, (ii) an LSTM layer incorporating dropout for regularisation, and (iii) a dense layer with a sigmoid activation function to produce binary classification output.

##### Bidirectional Long Short-Term Memory (Bi-LSTM)

Bi-LSTM networks consist of two LSTM networks, in which one feeds the data in a forward direction, while the other feeds the data in a backward direction [35]. We used Keras [34] library for implementing the model layers. Our architecture consisted of the following components: (i) an embedding layer for word representation, (ii) a Bi-LSTM layer incorporating dropout for regularisation, and (iii) a dense layer with a sigmoid activation function to produce binary classification output.

##### Convolutional Neural Network (CNN)

CNN adaptively learns the different hierarchies of features through back propagation using different layers such as convolution layers, pooling layers and fully connected layers [36]. Even though convolutional networks were initially developed by the neural network image processing community where it excelled in recognising objects in predefined classes, it has recently shown excellent outcomes in NLP tasks, especially in sentence classification into predefined categories. CNN and extended CNN architectures [37] have been successfully applied to text classification tasks of different granularity [38]. They also capture the neighbourhood relation via the window size of the CNN filter. We used the Keras [34] library for implementing the model layers. Our model architecture includes the following components: (i) an embedding layer for word representation, (ii) a 1-Dimensional convolutional layer with multiple filters and rectified linear unit (ReLU) activation, (iii) a global max pooling layer to capture relevant features and (iv) a dense layer with a sigmoid activation for binary classification.

### Text preprocessing and representation

TF-IDF (Term Frequency-Inverse Document Frequency) vectorisation converts the text data into numerical features in SVM and RF. Text data preprocessing, without the removal of stop words, includes tokenisation and sequence padding. For LSTM, Bi-LSTM and CNN models, we tokenised the text using the Keras [34] tokenizer with a specified maximum word count. To ensure that we capture the full context without unnecessary truncation, we selected a maximum token length of 30. This choice is well-justified, as it allows us to handle all sequences within our dataset, thereby capturing the most of the context and information from each text entry, without disclosing any patient-specific information.

In order to enhance the representational capacity and capture intricate patterns in our data across various machine learning models, our classical ML models with TF-IDF vectorization has a dimension within a range of 2450 to 2520 for the applied random state values, and we choose 300-dimensional input vectors consistently in all our DL models, following current practice [39]. Unlike some deep learning models that have fixed dimensions, TF-IDF vectors adapt their dimensionality based on the dataset’s linguistic diversity. TF-IDF captures word significance across documents, while fixed dimensions in deep learning aim for computational efficiency and concise representations.

### Hyperparameter tuning

For each model, hyperparameter tuning is performed using grid search [40] and five-fold cross-validation. The goal is to find the optimal set of hyperparameters for each model. For SVM, various combinations of hyperparameters, including ‘C’ (regularization parameter), ‘kernel’ (kernel function), and ‘gamma’ (kernel coefficient) were tried. For RF, combinations of ‘n_estimators’ (the number of trees in the forest), ‘max_depth’ (the maximum depth of the trees), and ‘max_features’ (the number of features to consider when splitting nodes) were used. For LSTM and Bi-LSTM we combined the hyperparameters units, dropout and recurrent dropout. Finally, for CNN, hyperparameters, such as filters and kernel size were selected using grid search. In LSTM, Bi-LSTM and CNN models cross entropy was used as the loss function, adamax as the optimizer, early stopping as the stop criteria and run with an epoch value of ten. Model architectures with the best hyperparameters are summarised in Table 2.

**Table 2:** Parameters used in different machine learning models after grid search optimization

Classifier	Parameters - Values
SVM	vectorisor - TF-IDF vectorisor (in a range of 2450 to 2520 dimensions)
	kernel - rbf (Radial Basis Function) kernel
	regularisation parameter - C value of 10
	cross-validation - 5 fold
RF	vectorisor - TF-IDF vectorisor (in a range of 2450 to 2520 dimensions)
	maximum features- square root of the total number of features
	number of decision trees - 100
	cross-validation - 5 fold
LSTM & Bi-LSTM	Embedding layer - 300 dimensional
	LSTM / Bi-LSTM layer - 128 nodes
	dropout and recurrent dropoutlayer -probability of 0.2
	dense output layer - 1 node
	activation layer - sigmoid
	cross-validation - 5 fold
	loss - binary cross entropy
	optimizer - adamax
CNN	Embedding layer - 300 dimensional
	1D convolutional layer with:
	- filters - 256
	- window size - 5
	- activation layer - relu
	dropout layer - probability of 0.5
	dense output layer - 1 node
	activation layer - sigmoid
	cross-validation - 5 fold
	loss - binary cross entropy
	optimizer - adamax

### Model assessment and selection

For each iteration through the random state values, we trained the classifiers with the best hyperparameters identified during the tuning phase. The performance was evaluated using precision, recall, and F1-score. We calculated the mean and standard deviation of these performance metrics across the different iterations to assess the overall model performance. The model with the highest F1 score was selected for further analysis.

### Visualisation

To gain insights into the distribution of the data in a lower-dimensional space, we applied t-distributed Stochastic Neighbour Embedding (t-SNE) [41] to the TF-IDF vectors of the test data in SVM and RF. For the DL models, the word embeddings learned by the best model are extracted and visualised. The resulting 2D scatter plot visualises the data points based on their predicted labels (‘y_test‘) and serves as an additional tool for understanding the model’s behaviour. t-SNE is a technique commonly used to explore intricate patterns and relationships within complex datasets by projecting them into a lower-dimensional space. This reveals hidden insights not apparent in the original data. t-SNE visualisations also offer an intuitive way to comprehend model performance, decision boundaries, and data separability. These are particularly popular for visualising text data due to their ability to capture complex relationships in high-dimensional data, making t-SNE a preferred choice to linear techniques, like Principal Component Analysis (PCA). However, one must be aware of the limitations of t-SNE, such as sensitivity to the perplexity parameter and difficulty in interpreting distances in the reduced space.

### Model explanation using LIME

After determining the most effective model, we perform feature relevance analysis to understand which terms or features have the greatest impact on the classification result. This analysis provides valuable insights into the key phrases or structures from which the model creates its predictions. To this end, we use a method called LIME [42], suited for predictions of complex black-box models. LIME starts by creating variations of the input text, involving actions like removing, replacing, or rearranging tokens randomly. It then passes these variations through the model and records the resulting predictions. LIME selects a subset of token features from both the original input text and the variations, focusing on those that significantly influence prediction. A linear SVM is then built using these selected features, which helps estimate the model’s behaviour regarding specific features. The feature importance weights calculated by this process clarify how the model’s output class is determined, highlighting the most influential tokens for predicted class probabilities. Higher weights signify stronger contributions, while lower weights indicate less influence. The application of LIME on machine learning models assists non-experts in comprehending the internal processes of a model and tracking decision details related to predictions.

## Results

### Classifier results

The model is optimised using the training data, and the chosen hyperparameters via grid search are then implemented in the model and analysed in the test performance. Performance metrics for each model were calculated in terms of precision (P), recall (R) and F1-score (F1) as shown in Table 3, with the different classifiers producing comparable good results. We opt for the F1-score instead of ROC and AUC, because of its better handling of imbalanced data and its alignment with the substantial clinical impact of both false positives and false negatives.

**Table 3:** Performance metrics for SVM, RF, LSTM, Bi-LSTM, and CNN models on the test data and their average prediction time per sample

Classifier	Metric	Mean $\varvec{\pm }$ Std Error	**95%** Confidence Intervals	Average Prediction Time (seconds)
SVM	P	$0.955 \pm 0.002$	$[0.951 - 0.959]$	0.258
	R	$0.955 \pm 0.002$	$[0.951 - 0.959]$	
	F1	$0.955 \pm 0.002$	$[0.951 - 0.959]$	
RF	P	$0.942 \pm 0.003$	$[0.936 - 0.948]$	0.057
	R	$0.942 \pm 0.003$	$[0.936 - 0.948]$	
	F1	$0.942 \pm 0.003$	$[0.936 - 0.948]$	
LSTM	P	$0.948 \pm 0.002$	$[0.944 - 0.952]$	0.501
	R	$0.948 \pm 0.002$	$[0.944 - 0.952]$	
	F1	$0.948 \pm 0.002$	$[0.944 - 0.952]$	
Bi-LSTM	P	$0.944 \pm 0.003$	$[0.938 - 0.950]$	0.502
	R	$0.946 \pm 0.003$	$[0.938 - 0.950]$	
	F1	$0.944 \pm 0.003$	$[0.938 - 0.950]$	
CNN	P	$0.954 \pm 0.002$	$[0.950 - 0.958]$	0.130
	R	$0.954 \pm 0.002$	$[0.950 - 0.958]$	
	F1	$0.954 \pm 0.002$	$[0.950 - 0.958]$	

Furthermore, classifier models were profiled with their computational speed assistance by identifying performance bottlenecks and enhancing the underlying hardware or software infrastructure to acquire faster execution times. The computational speed for each sample is estimated using the Python module “time”^3^ to measure the model prediction time. We calculated the performance of classifier models using an AMD Ryzen7 5700U with Radeon Graphics processor with a clock frequency of 1.8 GHz and 8 GB of RAM. The experiments were conducted using Python 3.8.16 running on Windows 11. Table 3 lists the mean prediction time per sample for each of the best models.

### t-SNE visualisation

**Fig. 2:**
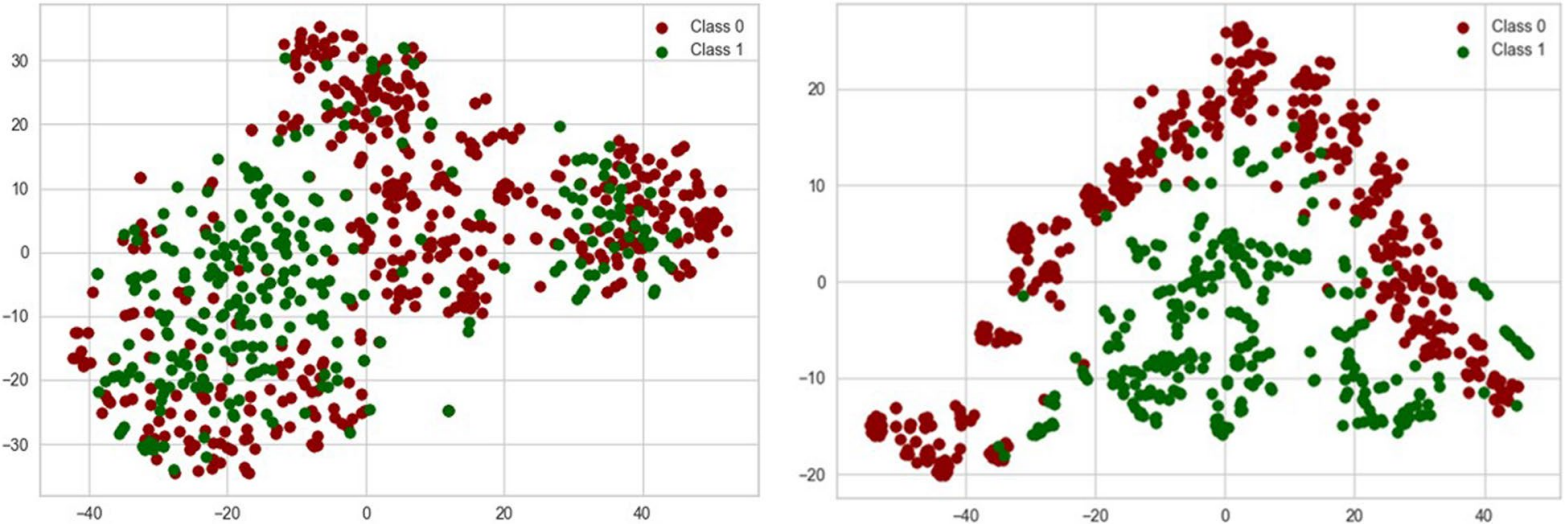
Visualization of vector representations of test data using t-SNE. (i) Static TF-IDF weighted vectors with no clear separability among the classes and (ii) Dynamic embedding representation showing a better separability among the classes

The input representation for the SVM is a high dimensional vector based on token occurrence, which remains static during training and can lead to reduced separability in the dimension-reduced visualisation. While the embeddings in the CNN model adapt to the downstream task, optimising their representation for the domain-specific task, enabling separability in the visualization. The grouping into corresponding class clusters is therefore recognisable in the embedding case for CNN but less clear for the SVM representation. Figure 2 plots the visualisation of test data using the t-SNE method for the SVM and the CNN model. In summary, the t-SNE visualisation shows that the dynamic embedding approach of the CNN results in a better separability of data clusters compared to the static representation of SVM, highlighting the adaptability of neural networks in domain-specific tasks.

### LIME explanation

**Fig. 3:**
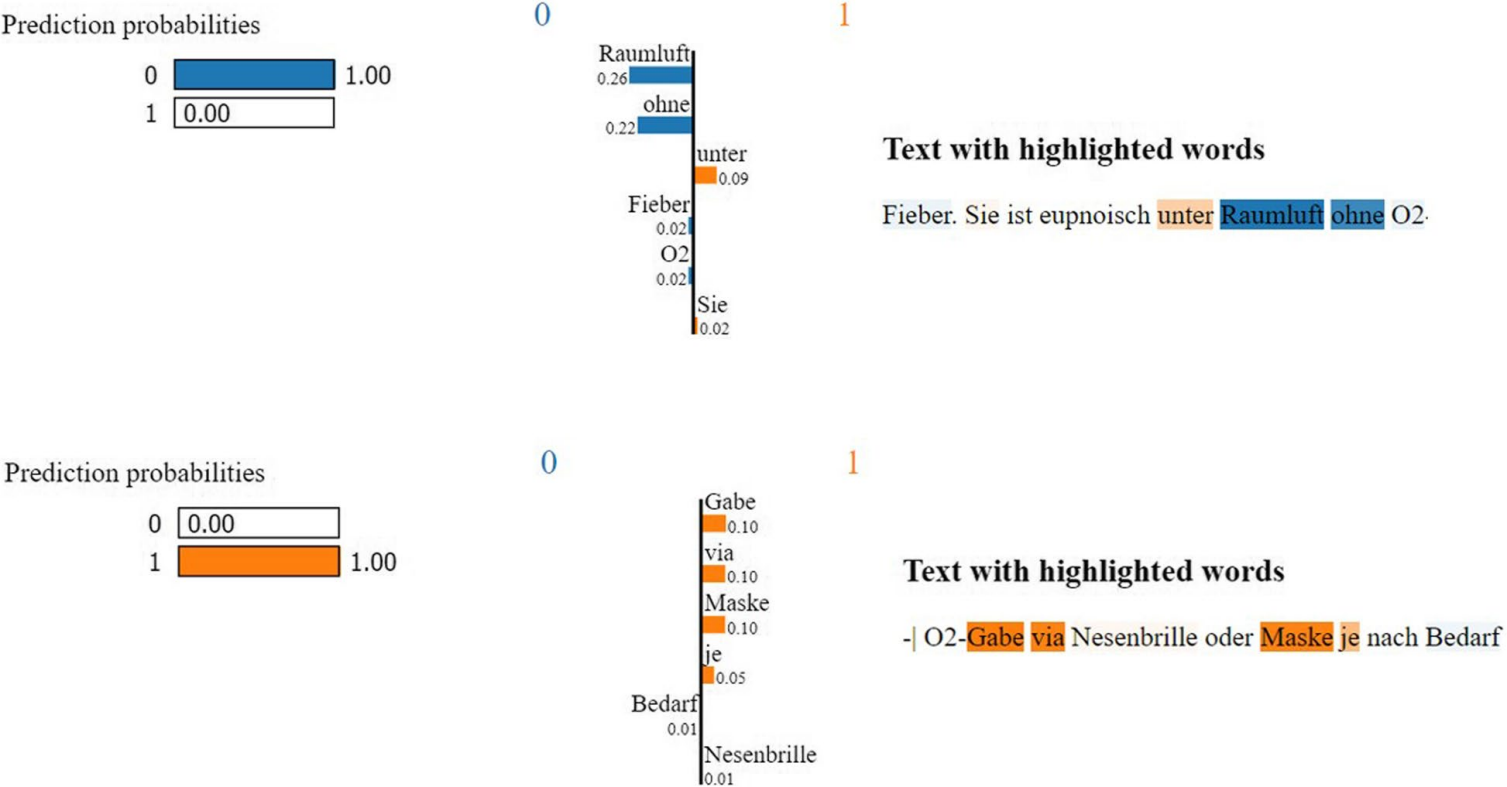
LIME providing insights into model predictions by highlighting the key tokens influencing the classification decision

LIME explanations are generated to give insight into the model’s prediction. Figure 3 illustrates the LIME explanation for the SVM model prediction for both class “0” and class “1” on specific text lines. LIME identified the most influential tokens contributing to the model prediction. The weights of each of the influential tokens are sorted based on their class predictions, and the sum of the weights for each class is calculated to reach the predicted class [43]. Tokens in input text are highlighted based on their probabilities of falling into a class. These explanations contribute significantly to understanding the decision-making process [44].

In our comprehensive analysis of top tokens using LIME for both SVM and CNN models, we gained granular insights into the differentiating features that determine classification performance. This not only enhances our understanding of model predictions but also provides interpretability, shedding light on the key factors influencing the decision-making process within these complex models. The most important tokens for this dataset according to our analysis were “raumluft”, “kein”, “nicht”, “ohne” for class 0 (no supplemental oxygen) and “FiO2”, “mit”, “gabe”, numbers followed by “L” or “l” determining the litres for with supplemental oxygen (class 1), cf. Table 4 for the top 10 tokens per class.

**Table 4:** Top influential tokens in the dataset for SVM and CNN models using LIME

Ranking	Class 0		Class 1	
	SVM	CNN	SVM	CNN
1	raumluft	kein/keine	l/L/Litre	fiO2/FiO2
2	akuter	ohne	fiO2/FiO2	l/L/Litre
3	Kein/kein/keinem	via	Flüssigsauerstofftherapie	Mit
4	ohne	raumluft	pflichtig	Gabe
5	auszugehen	AF	Zufuhr	O2
6	nicht	mmol	Gabe	Flow
7	Pulsoxy	nicht	inadäquaten	Sauerstoff
8	Aufsättigung	K	mehr	für
9	niedriger	zufuhr	Darunter	Brille
10	ausgeprägter	seit	Mit	wurde

### Error analysis

Confusion matrices are designed to give the predicted values in a count format, which distinguishes between correct and incorrect predictions. The true positive and true negative values provide a clear picture of the correct predictions within the network, while the false positives and false negatives are the topics of interest for error analysis.

**Fig. 4:**
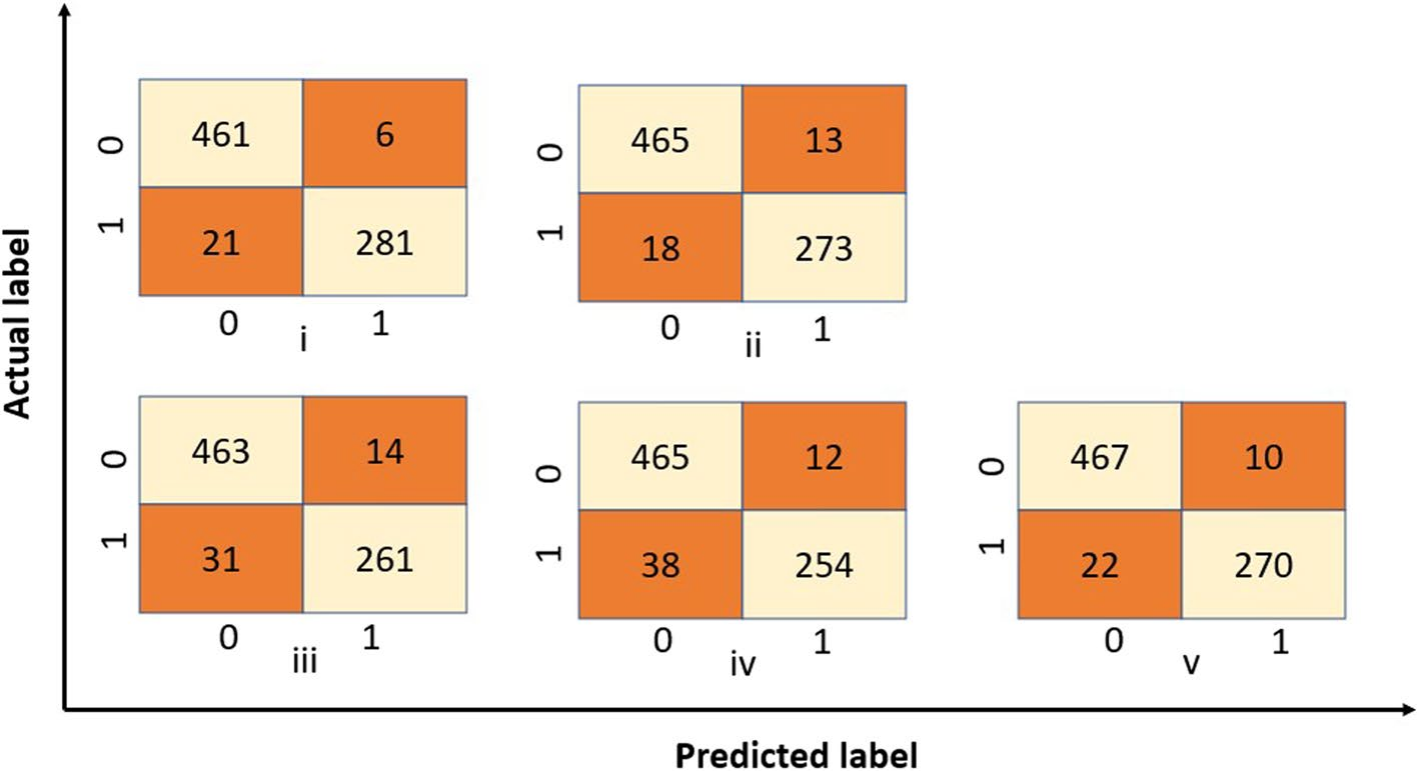
Confusion matrices depicting the performance of (i) SVM, (ii) RF, (iii) LSTM, (iv) Bi-LSTM, and (v) CNN models

Analysing the false positives, i.e. the number of incorrectly assigned text lines to have received oxygen supplementation and false negatives, i.e. the number of incorrectly assigned text lines to not have received oxygen supplementation, it was of interest that for all random state values, there were overlapping texts in these categories within all models, i.e., for a random state value of 729 there were 7 and 8 overlapping false positive and false negatively classified texts in all models, cf. Table 5. Figure 4 illustrated the values obtained for the confusion matrices for different models at the random state value of 729.

**Table 5:** Common text segments identified as false positives (FP) and false negatives (FN) during error analysis of the test data

FP/FN	Text span
FP	“O2 Sättigungswert vom 1.12.2019 mit Normalwerten”
	“O2 saturation value from 1.12.2019 with normal values”
FP	“respiratorischem Infekt und keinem erhöhten O2 Bedarf”
	“respiratory infection and no increased O2 requirement”
FN	“SO2-Bedarf”
	“SO2 requirement”
FN	“Laut Pflegebericht: im PH trotz Sauerstoffgabe Sättigung von 65% und”
	“According to the care report: saturation of 65% in the PH despite oxygen administration and”

## Discussion

In all languages, but particularly in languages other than English, the access to comprehensive clinical narrative datasets for public use poses a challenge. Legal restrictions, privacy policies, and stringent data protection regulations limit their availability and hinder their publication [45]. Unfortunately, this constrained environment has impeded our ability to test our models on diverse datasets, thereby limiting our capacity to confirm the generalizability of our findings. Consequently, faced with these limitations, we opted to create our dataset for the experiment. The process of manual annotation proved to be a particularly arduous task, underscoring the challenges associated with compiling and annotating clinical narrative data under such restrictions.

Since the dataset is imbalanced with a substantially higher number of text snippets in class “0” compared to class “1”, accuracy is not a suitable metric for evaluating model performance. Hence, precision, recall and F1-score are more informative as they provide a better measure of the model’s potential to detect the minority class. Table 3 highlights that the performance of classical ML models overlaps with DL models. Despite the emergence of DL models, there are several applications where classical ML such as SVM outperformed DL approaches [46]. Even image classifiers related to the COVID-19 context have observed this phenomenon [47, 48].

In addition to the model’s predictive performance, the mean prediction time per sample was also assessed. Out of this assessment, the classical ML model (RF) is the fastest network for this text classification task. In comparison with Saadatmand et al. [13] and Yamanaka et al. [14], who used certain features such as demographics, symptoms, patient background, etc. for determining the requirement of oxygen therapy, our experiments were especially focused on clinical narratives for oxygen status. In contrast to Muto et al. [16], which relies on decision support from clinicians, our methodology leverages an explainable AI module to understand the model decisions.

Even Fig. 2 does not show clear and distinct clusters for CNN as one class appears as an inverted V shape, while the other class is spread inside, which suggests that the classes might not be easily separable in the embedded space. This reveals the possibility of (i) overlap between classes, (ii) high intrinsic dimensionality that may not be captured by t-SNE, and (iii) complex non-linear relationships within the data.

## Conclusion

In this paper, text lines extracted from German-language discharge summaries of COVID-19 patients were used to detect patients who received supplementary oxygen therapy, which constitutes important information for building cohorts for retrospective COVID-19 clinical studies. The classification task had to distinguish the mention of oxygen related to oxygen measurement from the mention of oxygen in the context of oxygen supplementation.

Of the applied classification methods using classical machine learning to deep learning models, the performance of all of them (SVM, RF, LSTM, Bi-LSTM, and CNN) was similar. When comparing their computational efficiency, the RF model stood out, being the fastest classifier for this task, as well as in terms of training efforts. LIME aided in analysing and explaining the model predictions and played a crucial role in understanding the model performance. The pandemic highlighted the need for computerised classifications for the effective management of patient information in hospitals and for clinicians.

In future work, we aim to expand our research by acquiring additional datasets, thereby enhancing the robustness and generalizability of our model. We also plan to investigate its performance across diverse languages, ensuring its applicability and effectiveness in a broader linguistic context.

### Supplementary Information

**Additional file 1.**

## Data Availability

The datasets generated and analysed during this study are not publicly available in accordance to the local ethics approval. However, they can be made accessible from the corresponding author in consultation with the institutional review board of the Medical University of Graz on reasonable request.

## Abbreviations

COVID-19: Coronavirus disease 2019
SARS-CoV-2: Severe acute respiratory syndrome coronavirus
FiO2: Fraction of inspired oxygen
PaO2/PO2: Partial pressure of oxygen
SaO2: Arterial oxygen saturation
SpO2: Peripheral oxygen saturation
NLP: Natural Language Processing
SVM: Support vector machine
SVC: Support vector classifier
LSTM: Long short-term memory
Bi-LSTM: Bidirectional LSTM
CNN: Convolutional neural network
RF: Random Forest
EHRs: Electronic health records
NER: Named entity recognition
NEN: Named entity normalization
ANN: Artificial neural networks
RNN: Recurrent neural network
LIME: Local interpretable model-agnostic explanations
XAI: Explainable artificial intelligence
DL: Deep learning
ML: Machine learning
t-SNE: t-distributed Stochastic Neighbour Embedding
TF-IDF: Term frequency-inverse document frequency
PCA: Principal component analysis

## Footnotes

1
SO%|[sS][apP]?[O0o][2 ^2^ ](?!.)|bO2.?S ä |bO2(?!.*?pH)|[Ss] ä ttigung.

2 [509, 906, 331, 172, 729, 250, 762, 629, 926, 392]

3
https://docs.python.org/3/library/time.html
