# Supplementary material for: O2 supplementation disambiguation in clinical narratives to support retrospective COVID-19 studies
Source: BMC Med Inform Decis Mak. 2024 Jan 31;24:29. doi: 10.1186/s12911-024-02425-2 (PMC10829265; doi:10.1186/s12911-024-02425-2)
Supplement: Supplementary file 1 — Additional file 1. [file 12911_2024_2425_MOESM1_ESM.pdf]

Supplementary Material

1 Grid Search Parameters

In this section, we present the detailed results of the grid search performed to optimize hyperparameters for the Support Vector Machine (SVM), Random Forest (RF), Long Short Term Memory (LSTM), Bidirectional Long Short Term Memory (Bi-LSTM) and Convolutional Neural Network (CNN) models. Grid search is a systematic approach used to explore a range of hyperparameter values, helping to identify the combination that yields the best model performance. The trialed parameters and the cross validation scores are detailed in the table with their ranking. We list below only the best random state value for data split in each model (see Table s1, s2, s3).

Support Vector Machine (SVM)

For the SVM model, we conducted a grid search over the following hyperparameters:

- C (Regularization Parameter): [0.1, 1, 10]
- Kernel: [linear, rbf]
- Gamma: [scale, auto, 0.001, 0.01, 0.1, 1]

Random Forest (RF)

For the RF model, we conducted a grid search over the following hyperparameters:

- n_estimators: [10, 50, 100],
- max_depth: [None, 10, 20],
- max_features: [sqrt, log2]

Long Short Term Memory (LSTM) and Bidirectional Long Short Term Memory (Bi-LSTM)

For the LSTM and Bi-LSTM models, we conducted a grid search over the following hyperparameters:

- units: [64, 128],
- dropout: [0.2, 0.5],
- recurrent_dropout: [0.2, 0.5]

Convolutional Neural Network (CNN)

For the CNN model, we conducted a grid search over the following hyperparameters:

- filters: [64, 128, 256],
- kernel_size: [3, 5]

2 Analysis of False Positives and False Negatives

Understanding the instances where our models exhibit false positives and false negatives is crucial for refining their performance. In this section, we present an in-depth analysis of overlapping misclassifications across various models, shedding light on common challenges and areas for improvement for a given random state value.

Common False Positives

Table s4 outlines instances where all models produced false positives. These are cases where the models incorrectly predicted a positive class label, despite the ground truth being negative.

Common False Negatives

Similarly, Table s4 also presents instances where all models shared false negatives. These are cases where the models incorrectly predicted a negative class label, while the ground truth was positive.

Figure s1 illustrates the overlapping false positive and false negatives from the confusion matrix for a random state value of 729. The number of overlapping false positives and false negatives are highlighted at the center, while the remaining false positives and false negative values (actual value-overlapping value) are listed on each of the model wings within the digram.

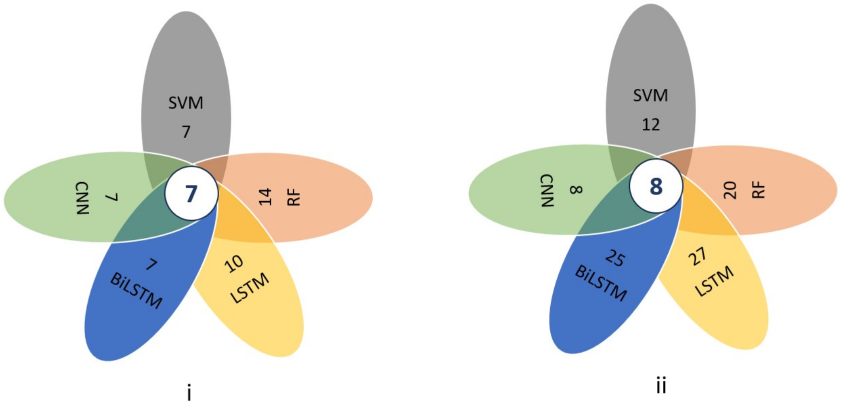

Figure s1: (i) False positive and (ii) false negative intersections between all 5 models in random state splitting at a value of 729.

mean_fit_time	std_fit_time	mean_score_time	std_score_time	params	split0_test_score	split1_test_score	split2_test_score	split3_test_score	split4_test_score	mean_test_score	std_test_score	rank_test_score
0.679	0.005	0.147	0.002	'C': 0.1, 'gamma': 'scale', 'kernel': 'linear'	0.876	0.875	0.859	0.875	0.867	0.870	0.007	20
0.823	0.014	0.207	0.005	'C': 0.1, 'gamma': 'scale', 'kernel': 'rbf'	0.754	0.727	0.732	0.730	0.724	0.733	0.011	26
0.676	0.005	0.146	0.005	'C': 0.1, 'gamma': 'auto', 'kernel': 'linear'	0.876	0.875	0.859	0.875	0.867	0.870	0.007	20
0.761	0.008	0.199	0.003	'C': 0.1, 'gamma': 'auto', 'kernel': 'rbf'	0.629	0.629	0.628	0.628	0.628	0.628	0.001	31
0.675	0.006	0.147	0.003	'C': 0.1, 'gamma': 0.001, 'kernel': 'linear'	0.876	0.875	0.859	0.875	0.867	0.870	0.007	20
0.774	0.014	0.196	0.004	'C': 0.1, 'gamma': 0.001, 'kernel': 'rbf'	0.629	0.629	0.628	0.628	0.628	0.628	0.001	31
0.677	0.006	0.144	0.005	'C': 0.1, 'gamma': 0.01, 'kernel': 'linear'	0.876	0.875	0.859	0.875	0.867	0.870	0.007	20
0.786	0.003	0.201	0.001	'C': 0.1, 'gamma': 0.01, 'kernel': 'rbf'	0.629	0.629	0.628	0.628	0.628	0.628	0.001	31
0.676	0.007	0.146	0.002	'C': 0.1, 'gamma': 0.1, 'kernel': 'linear'	0.876	0.875	0.859	0.875	0.867	0.870	0.007	20
0.803	0.007	0.205	0.003	'C': 0.1, 'gamma': 0.1, 'kernel': 'rbf'	0.655	0.654	0.646	0.654	0.646	0.651	0.004	30
0.674	0.004	0.145	0.004	'C': 0.1, 'gamma': 1, 'kernel': 'linear'	0.876	0.875	0.859	0.875	0.867	0.870	0.007	20
0.835	0.012	0.209	0.002	'C': 0.1, 'gamma': 1, 'kernel': 'rbf'	0.754	0.727	0.732	0.730	0.724	0.733	0.011	26
0.495	0.007	0.102	0.008	'C': 1, 'gamma': 'scale', 'kernel': 'linear'	0.943	0.925	0.935	0.920	0.932	0.931	0.008	12
0.805	0.071	0.170	0.002	'C': 1, 'gamma': 'scale', 'kernel': 'rbf'	0.937	0.927	0.941	0.925	0.935	0.933	0.006	4
0.507	0.019	0.097	0.002	'C': 1, 'gamma': 'auto', 'kernel': 'linear'	0.943	0.925	0.935	0.920	0.932	0.931	0.008	12
0.788	0.010	0.200	0.003	'C': 1, 'gamma': 'auto', 'kernel': 'rbf'	0.629	0.629	0.628	0.628	0.628	0.628	0.001	31
0.490	0.009	0.096	0.001	'C': 1, 'gamma': 0.001, 'kernel': 'linear'	0.943	0.925	0.935	0.920	0.932	0.931	0.008	12
0.792	0.012	0.203	0.003	'C': 1, 'gamma': 0.001, 'kernel': 'rbf'	0.629	0.629	0.628	0.628	0.628	0.628	0.001	31
0.492	0.009	0.098	0.002	'C': 1, 'gamma': 0.01, 'kernel': 'linear'	0.943	0.925	0.935	0.920	0.932	0.931	0.008	12
0.801	0.006	0.206	0.002	'C': 1, 'gamma': 0.01, 'kernel': 'rbf'	0.683	0.667	0.662	0.672	0.659	0.668	0.009	29
0.489	0.008	0.098	0.002	'C': 1, 'gamma': 0.1, 'kernel': 'linear'	0.943	0.925	0.935	0.920	0.932	0.931	0.008	12
0.668	0.008	0.168	0.011	'C': 1, 'gamma': 0.1, 'kernel': 'rbf'	0.907	0.899	0.904	0.901	0.906	0.903	0.003	19
0.491	0.008	0.098	0.001	'C': 1, 'gamma': 1, 'kernel': 'linear'	0.943	0.925	0.935	0.920	0.932	0.931	0.008	12
0.775	0.066	0.171	0.004	'C': 1, 'gamma': 1, 'kernel': 'rbf'	0.937	0.927	0.941	0.925	0.935	0.933	0.006	4
0.452	0.012	0.075	0.004	'C': 10, 'gamma': 'scale', 'kernel': 'linear'	0.937	0.930	0.914	0.928	0.948	0.931	0.011	6
0.937	0.031	0.171	0.006	'C': 10, 'gamma': 'scale', 'kernel': 'rbf'	0.943	0.937	0.948	0.940	0.946	0.943	0.004	1
0.441	0.004	0.076	0.005	'C': 10, 'gamma': 'auto', 'kernel': 'linear'	0.937	0.930	0.914	0.928	0.948	0.931	0.011	6
0.810	0.018	0.204	0.006	'C': 10, 'gamma': 'auto', 'kernel': 'rbf'	0.629	0.629	0.628	0.628	0.628	0.628	0.001	31
0.440	0.004	0.071	0.001	'C': 10, 'gamma': 0.001, 'kernel': 'linear'	0.937	0.930	0.914	0.928	0.948	0.931	0.011	6
0.803	0.006	0.206	0.003	'C': 10, 'gamma': 0.001, 'kernel': 'rbf'	0.685	0.668	0.668	0.672	0.662	0.671	0.008	28
0.445	0.015	0.072	0.001	'C': 10, 'gamma': 0.01, 'kernel': 'linear'	0.937	0.930	0.914	0.928	0.948	0.931	0.011	6
0.649	0.014	0.158	0.001	'C': 10, 'gamma': 0.01, 'kernel': 'rbf'	0.912	0.901	0.907	0.902	0.915	0.908	0.006	18
0.439	0.004	0.071	0.002	'C': 10, 'gamma': 0.1, 'kernel': 'linear'	0.937	0.930	0.914	0.928	0.948	0.931	0.011	6
0.517	0.013	0.113	0.004	'C': 10, 'gamma': 0.1, 'kernel': 'rbf'	0.951	0.937	0.943	0.930	0.945	0.941	0.007	3
0.439	0.006	0.072	0.002	'C': 10, 'gamma': 1, 'kernel': 'linear'	0.937	0.930	0.914	0.928	0.948	0.931	0.011	6
0.902	0.009	0.170	0.004	'C': 10, 'gamma': 1, 'kernel': 'rbf'	0.943	0.937	0.948	0.940	0.946	0.943	0.004	1

Table s1: Best hyperparameters for SVM model on random split value of 762

mean_fit_time	std_fit_time	mean_score_time	std_score_time	params	split0_test_score	split1_test_score	split2_test_score	split3_test_score	split4_test_score	mean_test_score	std_test_score	rank_test_score
0.479	0.053	0.008	0.002	'max_depth': None, 'max_features': 'sqrt', 'n_estimators': 10	0.891	0.925	0.933	0.915	0.878	0.909	0.021	5
2.251	0.050	0.028	0.006	'max_depth': None, 'max_features': 'sqrt', 'n_estimators': 50	0.933	0.935	0.935	0.927	0.915	0.929	0.007	2
4.487	0.042	0.049	0.004	'max_depth': None, 'max_features': 'sqrt', 'n_estimators': 100	0.933	0.940	0.945	0.932	0.924	0.935	0.007	1
0.458	0.021	0.008	0.001	'max_depth': None, 'max_features': 'log2', 'n_estimators': 10	0.893	0.896	0.912	0.870	0.888	0.892	0.014	8
2.253	0.036	0.031	0.002	'max_depth': None, 'max_features': 'log2', 'n_estimators': 50	0.930	0.928	0.928	0.920	0.889	0.919	0.015	4
4.497	0.060	0.061	0.003	'max_depth': None, 'max_features': 'log2', 'n_estimators': 100	0.930	0.927	0.933	0.919	0.912	0.924	0.008	3
0.118	0.012	0.005	0.001	'max_depth': 10, 'max_features': 'sqrt', 'n_estimators': 10	0.816	0.862	0.782	0.797	0.798	0.811	0.028	12
0.537	0.004	0.018	0.002	'max_depth': 10, 'max_features': 'sqrt', 'n_estimators': 50	0.818	0.815	0.833	0.784	0.816	0.813	0.016	11
1.095	0.033	0.032	0.000	'max_depth': 10, 'max_features': 'sqrt', 'n_estimators': 100	0.820	0.849	0.891	0.833	0.829	0.844	0.025	10
0.071	0.002	0.006	0.002	'max_depth': 10, 'max_features': 'log2', 'n_estimators': 10	0.663	0.754	0.649	0.663	0.686	0.683	0.038	16
0.369	0.026	0.017	0.003	'max_depth': 10, 'max_features': 'log2', 'n_estimators': 50	0.649	0.633	0.647	0.655	0.637	0.644	0.008	17
0.693	0.040	0.032	0.002	'max_depth': 10, 'max_features': 'log2', 'n_estimators': 100	0.657	0.631	0.644	0.649	0.634	0.643	0.010	18
0.208	0.010	0.006	0.001	'max_depth': 20, 'max_features': 'sqrt', 'n_estimators': 10	0.883	0.881	0.898	0.855	0.862	0.876	0.015	9
1.019	0.033	0.019	0.001	'max_depth': 20, 'max_features': 'sqrt', 'n_estimators': 50	0.901	0.904	0.927	0.875	0.901	0.901	0.017	6
2.223	0.074	0.042	0.005	'max_depth': 20, 'max_features': 'sqrt', 'n_estimators': 100	0.904	0.919	0.919	0.881	0.883	0.901	0.016	7
0.132	0.008	0.007	0.001	'max_depth': 20, 'max_features': 'log2', 'n_estimators': 10	0.792	0.767	0.702	0.732	0.766	0.752	0.031	14
0.603	0.016	0.020	0.002	'max_depth': 20, 'max_features': 'log2', 'n_estimators': 50	0.750	0.766	0.748	0.754	0.767	0.757	0.008	13
1.220	0.044	0.042	0.007	'max_depth': 20, 'max_features': 'log2', 'n_estimators': 100	0.714	0.735	0.780	0.769	0.741	0.748	0.024	15

Table s2: Best hyperparameters for RF model on random split value of 906

model	mean_fit_time	std_fit_time	mean_score_time	std_score_time	params	split0_test_score	split1_test_score	split2_test_score	split3_test_score	split4_test_score	mean_test_score	std_test_score	rank_test_score
LSTM	9.252	0.175	0.312	0.008	'dropout': 0.2, 'recurrent_dropout': 0.2, 'units': 64	0.920	0.940	0.941	0.940	0.943	0.937	0.008	6
	12.060	0.348	0.334	0.022	'dropout': 0.2, 'recurrent_dropout': 0.2, 'units': 128	0.938	0.954	0.954	0.958	0.948	0.951	0.007	1
	10.127	1.277	0.316	0.014	'dropout': 0.2, 'recurrent_dropout': 0.5, 'units': 64	0.925	0.937	0.950	0.948	0.945	0.941	0.009	5
	12.164	0.621	0.327	0.009	'dropout': 0.2, 'recurrent_dropout': 0.5, 'units': 128	0.941	0.953	0.950	0.948	0.958	0.950	0.005	2
	9.168	0.361	0.377	0.144	'dropout': 0.5, 'recurrent_dropout': 0.2, 'units': 64	0.922	0.927	0.937	0.948	0.937	0.934	0.009	8
	12.041	0.301	0.393	0.142	'dropout': 0.5, 'recurrent_dropout': 0.2, 'units': 128	0.938	0.945	0.938	0.932	0.958	0.942	0.009	3
	9.267	0.166	0.316	0.009	'dropout': 0.5, 'recurrent_dropout': 0.5, 'units': 64	0.919	0.935	0.943	0.940	0.940	0.935	0.009	7
	12.072	0.343	0.341	0.017	'dropout': 0.5, 'recurrent_dropout': 0.5, 'units': 128	0.927	0.943	0.948	0.940	0.951	0.942	0.008	4
Bi-LSTM	14.509	0.306	0.404	0.021	'dropout': 0.2, 'recurrent_dropout': 0.2, 'units': 64	0.919	0.932	0.938	0.945	0.945	0.936	0.010	5
	19.749	0.144	0.414	0.017	'dropout': 0.2, 'recurrent_dropout': 0.2, 'units': 128	0.919	0.937	0.935	0.948	0.948	0.937	0.011	4
	14.503	0.310	0.406	0.022	'dropout': 0.2, 'recurrent_dropout': 0.5, 'units': 64	0.925	0.930	0.933	0.943	0.946	0.936	0.008	6
	19.747	0.147	0.427	0.026	'dropout': 0.2, 'recurrent_dropout': 0.5, 'units': 128	0.943	0.928	0.937	0.953	0.938	0.940	0.008	3
	14.521	0.332	0.401	0.018	'dropout': 0.5, 'recurrent_dropout': 0.2, 'units': 64	0.924	0.933	0.917	0.946	0.943	0.933	0.011	8
	19.833	0.180	0.409	0.005	'dropout': 0.5, 'recurrent_dropout': 0.2, 'units': 128	0.937	0.940	0.935	0.958	0.954	0.945	0.009	1
	14.692	0.204	0.405	0.015	'dropout': 0.5, 'recurrent_dropout': 0.5, 'units': 64	0.927	0.937	0.938	0.938	0.935	0.935	0.004	7
	19.876	0.211	0.409	0.006	'dropout': 0.5, 'recurrent_dropout': 0.5, 'units': 128	0.928	0.943	0.941	0.941	0.948	0.940	0.006	2
CNN	2.816	0.106	0.123	0.008	'filters': 64, 'kernel_size': 3	0.959	0.945	0.953	0.950	0.932	0.948	0.009	6
	3.396	0.060	0.121	0.011	'filters': 64, 'kernel_size': 5	0.953	0.946	0.953	0.959	0.946	0.952	0.005	4
	3.144	0.137	0.123	0.005	'filters': 128, 'kernel_size': 3	0.959	0.951	0.950	0.951	0.943	0.951	0.005	5
	4.042	0.086	0.129	0.003	'filters': 128, 'kernel_size': 5	0.961	0.951	0.950	0.954	0.953	0.954	0.004	3
	3.896	0.101	0.133	0.001	'filters': 256, 'kernel_size': 3	0.963	0.958	0.951	0.956	0.951	0.956	0.004	2
	4.922	0.159	0.140	0.003	'filters': 256, 'kernel_size': 5	0.958	0.959	0.951	0.961	0.954	0.957	0.004	1

Table s3: Best hyperparameters for LSTM model on random split value of 331, Bi-LSTM model on random split value of 509 and CNN model on random split value of 906

Text	Text	True Label	Predicted Label
FP	Befund Vorstellung mit Fragestelllung LTOT. Patient möchte keine Langzeit O2 Therapie, wenn nicht unbedingt nötig.,	0	1
	Dzt die klassische Indikation für Remdesivir nicht vorliegend (O2	0	1
	O2 Sättigungswert vom 1.12.2019 mit Normalwerten. Die aktuelle	0	1
	erforderlich machten. Im weiteren Verlauf wurde ab dem 20.12.2020 keine O2-Unterstützung mehr benötigt.	0	1
	Es erfolgte eine symptomorientierte Therapie. Eine O2 Therapie über die Nasenbrille war bei guten Blutgaswerten nicht	0	1
	respiratorischem Infekt (Indexpatientin?) und keinem erhöhten O2 Bedarf	0	1
	Die langsame Reorientierung mit der ausgeprägten cerebr. Mikroangiopathie und O2-Ensättigungen bis 80% iR. Des	0	1
FN	SO2-Bedarf	1	0
	Maske und Combivent-Inhalation kam es zu einem Anstieg der sO2 auf 91%	1	0
	Sauerstoffsättigungswerten erfolgte eine Flüssigsauerstofftherapie, darunter konnten stets suffiziente	1	0
	Bei durchwegs guten Sauerstoffsättigungswerten auch bei Raumluft kann die Sauerstofftherapie schließlich beendet.	1	0
	sich eine ausreichende Sauerstoffsättigung in Ruhe, eine Sauerstoffgabe ist nicht mehr vonnöten.	1	0
	sind. Die Patientin wir somnolent. Zuletzt steigert sich auch der Sauerstoffbedarf massiv, die Sauerstoffsättigung wird	1	0
	SO2 91 %. FI O2	1	0
	Laut Pflegebericht: im PH trotz Sauerstoffgabe Sättigung von 65%	1	0

Table s4: Overlapping False Positive (FP) and False Negative (FN) texts for all models in random state splitting at a value of 729
